# Formulation of Chlorine-Dioxide-Releasing Nanofibers for Disinfection in Humid and CO_2_-Rich Environment

**DOI:** 10.3390/nano12091481

**Published:** 2022-04-27

**Authors:** Barnabás Palcsó, Adrienn Kazsoki, Anna Herczegh, Ágoston Ghidán, Balázs Pinke, László Mészáros, Romána Zelkó

**Affiliations:** 1 University Pharmacy Department of Pharmacy Administration, Faculty of Pharmaceutical Sciences, Semmelweis University, Hőgyes Endre utca 7-9, H-1092 Budapest, Hungary; palcso.barnabas@pharma.semmelweis-univ.hu (B.P.); kazsoki.adrienn@pharma.semmelweis-univ.hu (A.K.); 2 Department of Conservative Dentistry, Faculty of Dentistry, Semmelweis University, Szentkirályi utca 47, H-1088 Budapest, Hungary; herczegh.anna@dent.semmelweis-univ.hu; 3 Institute of Medical Microbiology, Faculty of Medicine, Semmelweis University, Nagyvárad tér 4, H-1089 Budapest, Hungary; ghidan.agoston@med.semmelweis-univ.hu; 4 Department of Polymer Engineering, Faculty of Mechanical Engineering, Budapest University of Technology and Economics, Műegyetem rkp. 3, H-1111 Budapest, Hungary; pinke@pt.bme.hu (B.P.); meszaros@pt.bme.hu (L.M.); 5 MTA-BME Research Group for Composite Science and Technology, Műegyetem rkp. 3, H-1111 Budapest, Hungary

**Keywords:** electrospinning, nanofibers, poly(ethylene oxide), sodium chlorite, chlorine dioxide, antibacterial, disinfection

## Abstract

Background: Preventing infectious diseases has become particularly relevant in the past few years. Therefore, antiseptics that are harmless and insusceptible to microbial resistance mechanisms are desired in medicine and public health. In our recent work, a poly(ethylene oxide)-based nanofibrous mat loaded with sodium chlorite was formulated. Methods: We tested the chlorine dioxide production and bacterial inactivation of the fibers in a medium, modeling the parameters of human exhaled air (ca. 5% (*v/v*) CO_2_, T = 37 °C, RH > 95%). The morphology and microstructure of the fibers were investigated via scanning electron microscopy and infrared spectroscopy. Results: Smooth-surfaced, nanoscale fibers were produced. The ClO_2_-producing ability of the fibers decreased from 65.8 ppm/mg to 4.8 ppm/mg with the increase of the sample weight from 1 to 30 mg. The effect of CO_2_ concentration and exposure time was also evaluated. The antibacterial activity of the fibers was tested in a 24 h experiment. The sodium-chlorite-loaded fibers showed substantial antibacterial activity. Conclusions: Chlorine dioxide was liberated into the gas phase in the presence of CO_2_ and water vapor, eliminating the bacteria. Sodium-chlorite-loaded nanofibers can be sources of prolonged chlorine dioxide production and subsequent pathogen inactivation in a CO_2_-rich and humid environment. Based on the results, further evaluation of the possible application of the formulation in face-mask filters as medical devices is encouraged.

## 1. Introduction

In the age of antimicrobial resistance, eliminating pathogens such as bacteria or viruses remains a grueling challenge in public and clinical health care. Preventing infections is a vital aspect of any successful antimicrobial management program. With the help of broad-spectrum antiseptics and disinfectants, the overuse of antibiotics and antiviral substances can be reduced [1]. To maintain adequate antibiotic stewardship, the use of effective, relatively harmless antiseptics and disinfectants that are insusceptible to microbial resistance mechanisms is desired. However, the rise of antiseptic-resistant pathogens is also a long-known phenomenon, especially in the hospital environment [2]. In the case of two common antiseptic agents, chlorhexidine and octenidine, it has been shown that by increasing the usage of these substances in practice, a significant reduction in susceptibility can be observed in isolated *Staphylococcus aureus* strains [3]. Increased minimum inhibitory concentrations (MICs) for both antiseptics in *Staphylococcus* spp. strains have also been reported [4]. Several reports have also been published regarding the acquired microbial resistance against disinfectants [5,6,7]. Therefore, other alternative antimicrobial substances should be examined to address the emerging issue of the wide spread of antibiotic and antiseptic-resistant pathogens. Chlorine dioxide (ClO_2_) is a gaseous oxidizing agent with good water solubility, used as a disinfectant mostly in water treatment and the food industry. Due to its unique molecular structure, the reactivity of ClO_2_ is limited mainly to thiol-group-containing amino acids. Other organic macromolecules are also potential substrates of ClO_2_, but the reaction rate is several magnitudes lower than in the case of thiol groups. The result of the chemical reaction and the mechanism of action of ClO_2_ are the change in protein structure and subsequent loss of function. Several reports claimed the effectiveness of chlorine dioxide against all kinds of microbes, including bacteria, fungi, protozoa and viruses. The gaseous substance has a remarkable penetrating ability, enabling it to decontaminate areas considered impervious, such as biofilms [8,9,10,11,12,13]. The safety of ClO_2_ relies not on the difference in macromolecules or metabolism of pathogens and human cells but on the difference in their size and the protecting factors existing in human tissue. Bacteria and viruses can be susceptible to a given concentration of ClO_2_ that does not represent any harm to human or animal cells [14]. It has been shown in several animal studies that a certain level of ClO_2_ exposure does not do any damage to the examined animals [15,16]. Although chlorine dioxide convincingly satisfies the requirements of an ideal biocide, it has not become a widely used compound in everyday practice. Reports have already been published considering the use of ClO_2_ in the disinfection of hospital environments such as water systems, air, rooms and even ambulance vehicles [17,18,19,20]. However, in medical practice, the use of ClO_2_ is mainly limited to dental applications such as root canal irrigation and mouth rinse [21,22]. The relatively small use of chlorine dioxide can be due to its cumbersome transportation and storage. The gaseous substance cannot be transported due to safety issues; therefore, chlorine dioxide is usually generated in aqueous solution at the site of application. One of the most common methods of chlorine dioxide production is the decomposition reaction of sodium chlorite (NaClO_2_) in the presence of acid, which results in chlorine dioxide and other byproducts. The dissolved gas has high volatility that causes a rapid decrease in the concentration of the solution limiting its shelf life.

Polymer-based formulations represent promising solutions to the issues related to the practical use of ClO_2_. Using polymer-based viscous solutions, the residence time of ClO_2_ can be prolonged [23]. Electrospun nanofibers loaded with sodium chlorite produce ClO_2_ under acidic conditions. Due to their high porosity and surface area to volume ratio, they can serve as efficient production sites for chlorine dioxide. Such nanosystems can enable ClO_2_ gas production and liberation at the site of use [24]. Polyethylene oxide is a water-soluble polymer that is relatively easy to process by electrospinning [25]. Sodium chlorite is also water-soluble; thus, there is a good chance of producing sodium chlorite nanofibers based on a common solvent. In our recent work, we present a polymer-based, nanofiber-based formulation with ClO_2_-generating ability. The nanofiber-based formulation is subjected to morphological and antibacterial examination. The ClO_2_-generating ability and the rate of chlorine dioxide liberation were also evaluated.

## 2. Materials and Methods

### 2.1. Materials

Poly(ethylene oxide) (PEO, average M_w_ = 600,000 g·mol^−1^) was obtained from Sigma-Aldrich (Budapest, Hungary). We used analytical-grade sodium chlorite (NaClO_2_, Sigma-Aldrich, Budapest, Hungary) as the active ingredient. Distilled water was filtered on a 0.22 μm PES filter before preparing the precursor polymer solution. The materials did not undergo any further purification.

### 2.2. Precursor Polymer Solutions and Electrospinning

**Figure 1:**
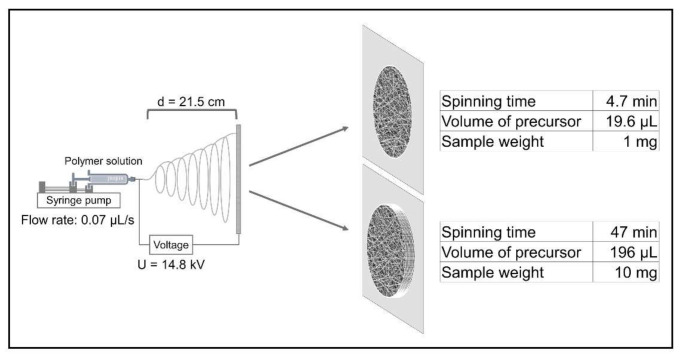
Electrospinning setup and preparation of two samples of different weights for illustration.

Aqueous polymer solutions were prepared by dissolving 5% (*w/w*) PEO in boiling water with continuous stirring. The samples were cooled down to room temperature and stirred overnight at 23 °C by magnetic stirring until homogenous solutions were obtained. Finally, sodium chlorite 20% (*w/w*) aqueous solution was added to the PEO solution to reach a concentration of 0.1% (*w/w*). Control PEO samples without active ingredients were prepared accordingly. Nanofiber production was carried out on lab-scale electrospinning equipment (SpinSplit Ltd., Budapest, Hungary). Precursor solutions were placed into plastic syringes (Luer lock, Sigma-Aldrich Ltd., Budapest, Hungary) with a volume of 3 mL and mounted to the pumping system of the instrument. The syringes were connected to a 22-gauge needle through Teflon tubes. The feeding rate for PEO control samples was 0.08 µL/s, with an applied voltage of 10.5 kV. The NaClO_2_-loaded PEO samples were prepared with a 0.07 µL/s feeding rate and 14.8 kV of applied voltage. The samples were collected on aluminum foil, and the needle-collector distance was set to 21.5 cm. The experiments were performed in a well-controlled room, the temperature was set to 23 ± 1 °C and the relative humidity was 25 ± 5% (Figure 1).

### 2.3. Fourier-Transform Infrared (FTIR) Spectroscopy

Infrared spectra of the components and the fibers were recorded with a Jasco FT/IR-4200 spectrophotometer, equipped with Jasco ATR PRO470-H single reflection accessory. Measurements were performed in absorbance mode, and spectra were collected over a wavenumber range of 4000 and 500 cm^−1^. For each spectrum, 100 scans were performed at a resolution of 4 cm^−1^. Spectra were evaluated with the software Spectra Manager-II (Jasco, Easton, MD, USA).

### 2.4. Scanning Electron Microscopy

Samples were fixed on a metal stub by conductive double-sided adhesive carbon discs and coated with a gold layer using a JEOL JFC-1200 Fine Coater (JEOL Ltd., Tokyo, Japan). Scanning electron microscopy (SEM) images were taken with a JEOL JSM-6380LA instrument (JEOL Ltd., Tokyo, Japan). The acceleration voltage and the working distance were 10 kV and 10 mm, respectively. For fiber diameter evaluation and distribution calculations, we measured the diameter of 100 random fibers using ImageJ (National Institutes of Health, Bethesda, MD, USA) software and prepared the histograms via Origin(Pro) software (Version 2018, OriginLab Corporation, Northampton, MA, USA).

### 2.5. Chlorine Dioxide Production of the Fibrous Samples

#### 2.5.1. Experimental Setup

The ClO_2_ production from NaClO_2_-loaded PEO fibers was carried out in 60 mL amber glass bottles. First, a 0.5 mL container was placed into the bottle, and 300 μL of distilled water was measured into the container to maintain a humid environment of RH > 95%. The water in the 0.5 mL container also served as a source for ClO_2_ concentration measurement. Next, the fibrous sample was stuck onto the inner wall of the bottle using double-sided adhesive tape, and CO_2_ was slowly injected into the glass to reach a concentration of 5, 10 or 15% (*v/v*). Bottles fitted with special caps, impermeable to ClO_2_, were kindly donated by Zoltán Noszticzius (Department of Physics, Budapest University of Technology and Economics, Budapest, Hungary). The suitable amount of CO_2_ gas was monitored and determined beforehand using a Ventis Pro5 multi-gas detector. Lastly, bottles were placed into an incubator and stored at 37 °C. To measure the concentration of chlorine dioxide generated from the fibers, the bottles were opened, and 100 μL was taken instantly from the distilled water placed in the 0.5 mL container. The 100 μL ClO_2_ aqueous solution was injected into a cuvette containing 2.9 mL of distilled water and measured using a Jasco V-750 UV-Visible spectrophotometer. The absorbance of the chlorine dioxide aqueous solution was recorded at 360 nm, where ClO_2_ has its characteristic absorption maximum. The molar absorptivity of ClO_2_ at 360 nm is 1250 cm^−1^ M^−1^ [26].

#### 2.5.2. Measurements and Data Analysis

To calculate the ClO_2_ concentration in the gas phase from concentrations measured in the aqueous solution, the following equation was used:

(1) $$c{\left(ClO_{2}\right)}_{g}=k\cdot K_{\theta }\cdot V_{m}\cdot c{\left(ClO_{2}\right)}_{aq}$$

where *k* is a constant, *K_θ_* is the distribution constant of ClO_2_, *V_m_* is the molar volume of the gas, *c*(*ClO*_2_)*_g_* is the chlorine dioxide concentration in the gas phase in ppm (μL/L) and *c*(*ClO*_2_)*_aq_* is the chlorine dioxide concentration in the aqueous solution in mol/L. At 37 °C, the value of the distribution constant is *K_θ_* = 0.053 and the molar volume is *V_m_* = 25.45 dm^3^/mol. The *k* constant is derived from the conversion of the units and its value is *k* = 1 × 10^6^ [27]. To evaluate the chlorine dioxide production of the samples the following measurements were carried out:

1. To measure the total amount of ClO_2_ generated in 24 h by fibers of various weights, we used 1, 5, 10, 15, 20 and 30 mg samples. Using these data, we calculated the ClO_2_-generating ability per weight and the ClO_2_ yield of the samples compared to the theoretical values. In this experiment, the CO_2_ concentration was set to 5%.
2. We examined the effect of different CO_2_ concentrations (5, 10, 15%) on the ClO_2_ production of 5 mg samples in a 24 h measurement.
3. The effect of residence time of fibers in the medium was evaluated by measuring the ClO_2_ production of 5 mg samples after 24, 48 and 72 h.

During all experiments, temperature was set to 37 °C, and RH was kept above 95%, as described above.

### 2.6. Bacterial Inactivation Study

To evaluate the antibacterial effect of the NaClO_2_-loaded PEO fiber mats, we used a nonhazardous bacterium, Enterococcus faecalis, as a model organism. Enterococcus faecalis was chosen due to its resilience and undemanding nature in terms of growth requirements, which ensured that bacterial inactivation could be attributed to the effect of the generated chlorine dioxide [28]. The setup and reaction conditions were analogous to those described in the ClO_2_ production study. Bacteria were added to the system via disposable inoculating loops, which were cut to make ca. 4 cm long pieces with the loop on their end. Each piece was glued into the inner part of the cap of an amber glass bottle, immersed into a bacterial suspension containing 2.8 × 10^9^ CFU/mL of E. faecalis, then sealed. Each inoculating loop contained approximately 1 μL of bacterial suspension. Sealed bottles were placed into an incubator and stored at 37 °C for 24 h. A medium similar to the composition of the air was prepared by setting the humidity and CO_2_ concentration in the glass containers to >95% and 5%, respectively [29]. After 24 h, we opened the bottles and immersed the inoculating loops into 100 μL of saline and stirred thoroughly to suspend the remaining bacteria. Finally, the entire 100 μL of suspension was streaked on a blood agar plate. The blood agar plates were incubated at 37 °C for another 24 h, and the surviving colonies were counted. Control samples with unloaded PEO fibers were also prepared accordingly.

## 3. Results

### 3.1. Morphological Characterization

**Figure 2:**
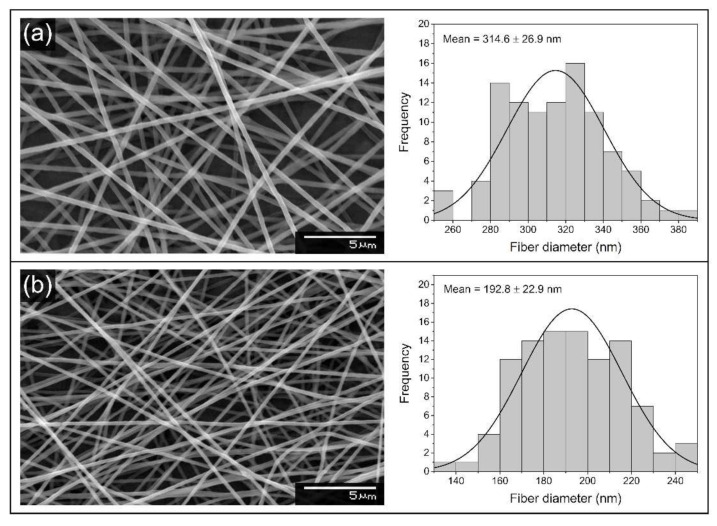
SEM images and histograms of the fiber diameter distribution of unloaded PEO (**a**) and NaClO_2_-loaded PEO (**b**) fibers.

Scanning electron microscopy images show randomly oriented, smooth-surfaced, nanoscale polymer fibers (Figure 2). Bead-like structures could not be observed throughout the samples. The average fiber diameter value for unloaded PEO fibers was 315 ± 27 nm, and the fiber diameters showed normal distribution (Figure 2a). Adding salt to the polymer solution and the subsequent changes in spinning parameters altered the average fiber diameter substantially, as the average fiber diameter value was 193 ± 23 nm in the case of NaClO_2_-loaded PEO fibers (Figure 2b). The fiber diameters of the NaClO_2_-loaded sample also showed normal distribution.

### 3.2. FTIR Analysis

**Figure 3:**
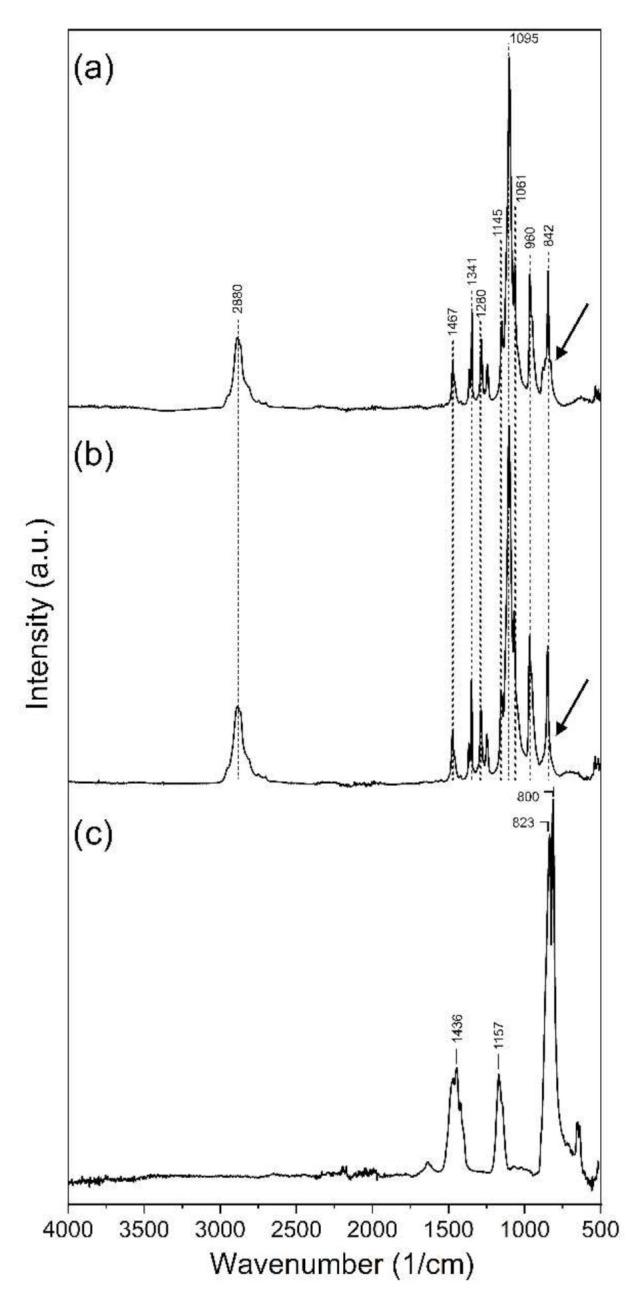
FTIR spectra of NaClO_2_-loaded PEO fibers (**a**), unloaded PEO fibers (**b**) and sodium chlorite (**c**).

To investigate the potential structural changes of the polymer macromolecules, FTIR spectra were recorded. Figure 3 shows the FTIR spectra of sodium chlorite, PEO and NaClO_2_-loaded PEO fibers. On the spectrum of PEO fibers, the peak at the band observed at 2890 cm^−1^ was assigned to the symmetrical C–H stretching (Figure 3b). The peaks at 1467, 1341, 1280 and 842 cm^−1^ represent the scissoring, wagging, twisting and rocking of the CH_2_ group. The sharp and intense band at 1095 cm^−1^, along with the peaks at 1145 and 1061 cm^−1^, is assigned to the asymmetric stretching vibration of the C–O–C bonds. The smaller, sharp peak at 960 cm^−1^ is due to C–C skeletal stretching vibrations [30]. The FTIR spectrum of the NaClO_2_-loaded fibers shows similar bands and intensities to that of the unloaded PEO sample (Figure 3a). A slight difference between the two spectra can be seen around the base of the peak at 842 cm^−1^ on the NaClO_2_-loaded PEO spectrum. Sodium chlorite has its sharp and intense peaks at 800 and 823 cm^−1^ (Figure 3c).

### 3.3. Chlorine Dioxide Production

**Figure 4:**
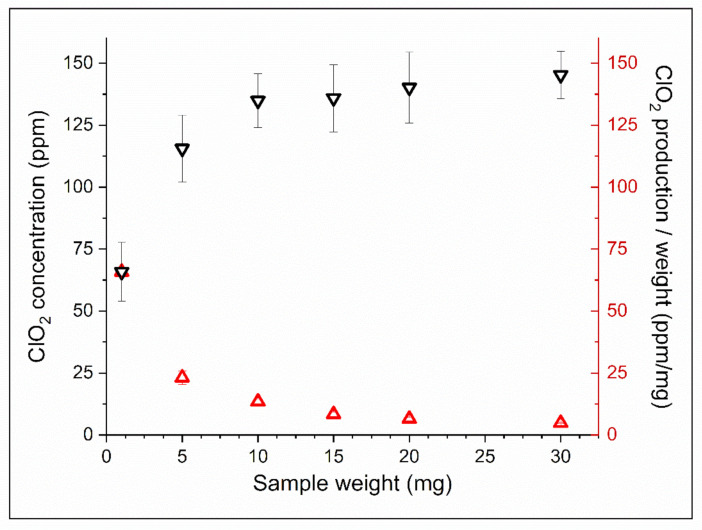
Chlorine dioxide production and chlorine dioxide production per weight of 1, 5, 10, 15, 20 and 30 mg samples.

The amount of chlorine dioxide generated by samples of different weights, along with the ClO_2_-producing ability per weight of these samples, is shown in Figure 4. When placing 1 mg of NaClO_2_-loaded PEO fiber into the medium, the ClO_2_ concentration in the gas phase reached 65.8 ppm (μL/L) after 24 h. Larger weights of samples resulted in higher ClO_2_ concentrations, but the rate of growth of the generated ClO_2_ remained substantially lower than expected. There was no significant difference in the produced sodium chlorite of samples of higher weights. The ClO_2_ production per weight data, however, show a gradual decrease in the ClO_2_ generation ability with increasing fiber weights, along with the yield of generated ClO_2_ compared to the theoretical values calculated from the NaClO_2_–acid reaction [31]. During the 24 h experiment, the ClO_2_ production ability and yield of 1 mg samples reached 65.8 ppm/mg and 89%, respectively, while the 30 mg samples could produce only 4.8 ppm/mg and 6.58% of the potential ClO_2_ yield (Figure 4, Table 1).

**Figure 5:**
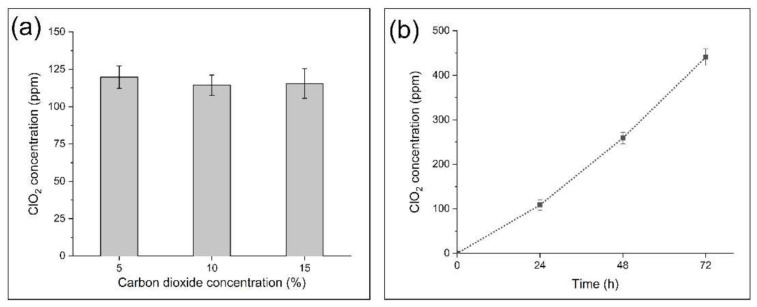
Effect of carbon dioxide concentration (**a**) and time (**b**) on ClO_2_-generating ability of 5 mg NaClO_2_-loaded PEO fibers.

Figure 5a shows the effect of carbon dioxide concentration on the ClO_2_ production ability of the NaClO_2_-loaded PEO fibers. We placed 5 mg of NaClO_2_-loaded PEO samples into amber glass bottles containing 5, 10 and 15% (*v/v*) of CO_2_, while humidity was kept above 95% and the temperature was set to 37 °C. After 24 h, there was no substantial difference in the produced ClO_2_. However, the time of exposure to CO_2_ in a humid environment resulted in significantly higher CO_2_ concentrations after 48 and 72 h (Figure 5b).

### 3.4. Bacterial Inactivation Study

**Figure 6:**
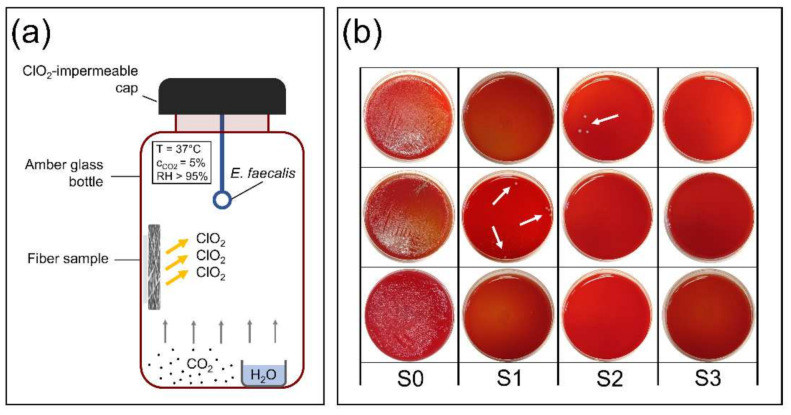
Experimental setup for bacterial inactivation study (**a**) and surviving colonies after incubation and counting (**b**) (S0: unloaded PEO fibers—control, S1: 1 mg NaClO_2_-loaded PEO, S2: 5 mg NaClO_2_-loaded PEO, S3: 10 mg NaClO_2_-loaded PEO fibers).

**Figure 7:**
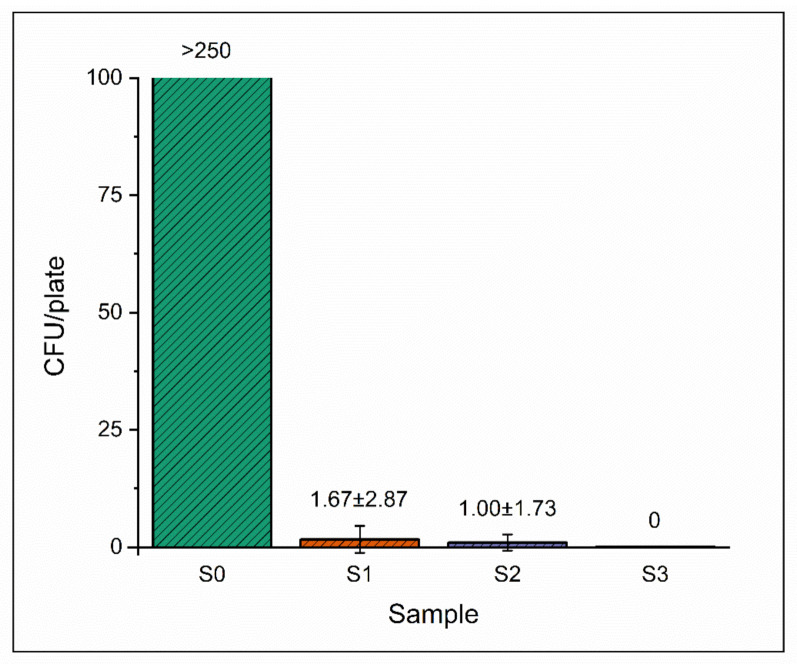
Average surviving colonies per plate of control (S0) and NaClO_2_-loaded PEO fibers (S1: 1 mg, S2: 5 mg, S3: 10 mg).

Figure 6a shows the setup of the bacterial inactivation experiment, while in Figure 6b, plates are shown after incubation and counting. We investigated the microorganism eliminating capacity of NaClO_2_-loaded PEO fibers of 1, 5 and 10 mg, along with unloaded PEO fibers. The average number of surviving colonies per plate is shown in Figure 7. In the case of unloaded PEO control samples, the number of colonies was too numerous to be counted in every parallel experiment, indicating that the reaction medium was optimal for bacterial growth (Figure 6b and Figure 7). As the weight of NaClO_2_-loaded PEO samples increased, the average survival decreased to zero. Only in one of the three parallels could surviving colonies be counted in the case of 1 and 5 mg NaClO_2_-loaded PEO samples, indicated by arrows (Figure 6b). Bacterial growth did not occur with 10 mg of NaClO_2_-loaded fibers.

## 4. Discussion

Scanning electron microscopy was used to evaluate the morphology of neat and NaClO_2_-loaded PEO-based samples prepared by electrospinning. The spinning process resulted in smooth and uniform fibers both in the case of unloaded PEO and NaClO_2_-loaded PEO samples; however, there was a substantial difference in the average fiber diameters. The applied voltage was 10.5 kV during the spinning of PEO fibers, which appeared to be insufficient when spinning NaClO_2_-loaded PEO samples; thus, the electric field needed to be increased. Generally, higher voltages result in lower fiber diameters, as the force pulling the fiber jet from the needle tip increases, resulting in the stretching and thinning of the ejected jet. However, studies have shown that the effect of higher voltages on fiber diameter may vary and depends on the composition of the precursor solution and other parameters of the electrospinning process [32,33]. The presence of inorganic salts increases the surface charge density and conductivity of the precursor polymer solution. The coulombic and electrostatic forces occurring between surface particles influence the stretching and thinning of the electrospun jet. Depending on the characteristics of the polymer macromolecule and the inorganic salt, the overall effect of the increasing salt concentration can result in either higher or lower average fiber diameter. In the case of polyvinylpyrrolidone (PVP), LiCl and MgCl_2_ had an increasing effect on fiber diameter, while NaCl decreased the average diameter [34]. The average fiber diameter of LiCl-loaded PEO nanofibers increased with higher salt concentrations [35]. After a slight decrease in the lower concentration regions, FeCl_3_ resulted in significantly thicker fibers when added to polyvinylidene fluoride. Lithium bromide, however, caused substantial thinning of the fibers when added to a styrene-based polymer system [36]. Figure 2 shows that NaClO_2_ present in the NaClO_2_-loaded PEO precursor solution and higher applied voltage together result in significantly thinner fibers compared to the PEO control sample.

Bands and intensities of the NaClO_2_-loaded PEO and unloaded PEO spectra are similar, except for the region around the peak at 842 cm^−1^, where a slight broadening can be observed on the NaClO_2_-loaded PEO spectrum (Figure 3a). Sodium chlorite has its most intense bands at 800 and 823 cm^−1^ (Figure 3c). Due to the significant (ca. 50-fold) difference in the concentrations of PEO and NaClO_2_, and the presence of the strong band at 842 cm^−1^ on the PEO spectrum, the intense peaks of NaClO_2_ are covered on the NaClO_2_-loaded PEO spectrum. The presence of NaClO_2_ in the fibrous sample causes only a minor broadening at the base of the peak at 842 cm^−1^ on the NaClO_2_-loaded PEO spectrum. Figure 3a,b also suggests that major changes did not occur in the structure and functional groups of PEO after loading NaClO_2_.

To evaluate the sodium chlorite generating ability of NaClO_2_-loaded PEO fibers, we placed samples into humid medium containing 5% (*v/v*) of CO_2_ and incubated them at 37 °C for 24 h. One of the common methods of ClO_2_ production is based on the reaction of sodium chlorite and acid (see Supplementary Materials). This phenomenon occurred in the fibrous mesh where carbonic acid, formed by water vapor and CO_2_ gas in situ, was the reactant. The high surface-to-volume ratio of nanofibers provides enough reaction sites for NaClO_2_ to form chlorine dioxide in the presence of H_2_CO_3_. A given portion of the generated chlorine dioxide dissolves in the excess of water placed in the 0.5 mL container. The concentration in the gas phase can be calculated from the data measured in the aqueous solution (see Supplementary Materials). In the case of the 1 mg NaClO_2_-loaded PEO sample, the yield was above 89% after 24 h of incubation (Table 1). However, higher amounts of NaClO_2_-loaded fibers did not result in proportionally higher ClO_2_ concentrations in the same experiment (Figure 4). This phenomenon could be attributed to the chemical reaction limited by the low concentration of carbon dioxide. Therefore, to test our hypothesis, we investigated the effect of higher CO_2_ concentrations on the ClO_2_-producing ability of the NaClO_2_-loaded fibers. The evaluation of the effect of carbon dioxide on ClO_2_ production showed that the limiting factor was not the concentration of the acidic reactant, as the ClO_2_ production did not change significantly after a two- and a three-fold increase in CO_2_ concentration (Figure 5a). It has also been shown that the time of exposure has a substantial impact on ClO_2_ production. Data suggest that the chlorine dioxide production in fiber samples is determined not only by the concentration of the reactants. When placing PEO nanofibers in a humid environment or fibers in contact with water directly, rapid dissolution and swelling of the mesh occur, resulting in a gel-like structure [37]. In our experiment, similar sizes were cut from the electrospun sample, but the thickness of the sample varied with spinning time (Figure 1). When preparing larger weights, the spinning time extended, resulting in an increase in the thickness of the fiber mesh. As swelling occurs in the outer layers of the sample, the fibrous texture disintegrates, the surface-to-volume ratio decreases and the gel-like layer acts as a barrier for diffusion, resulting in a reduced number of reaction sites and slower ClO_2_ production. The bacteria-eliminating ability of NaClO_2_-loaded PEO samples was tested in humid medium (RH > 95%) containing 5% CO_2_, similar to human breath. The overall ClO_2_ production in the 24 h experiment led to substantial bacterial inactivation with only slight deviations occurring in the average CFU/plate values of 1 and 5 mg samples. The inoculating loops placed into the bottles contained ca. 2.8 × 10^6^ CFUs, as their volume was 1 μL. The number of surviving colonies was too large to be counted regarding unloaded PEO fibers, while 1 and 5 mg of NaClO_2_-loaded PEO fibers reduced the average CFU/plate to 1.67 ± 2.87 and 1.00 ± 1.73 after 24 h, respectively. Chlorine dioxide emitted into the gas phase from samples weighing 10 mg killed all bacteria placed on the inoculating loops.

The dissolution and gelation of the nanofibers in humid media can be a source of prolonged ClO_2_ production; however, the structural changes may impair the usability of the fibrous mesh in certain conditions. The possible modifications and tailoring of the formulation by various polymers and compositions should be a matter of further investigation. A deeper understanding and investigation of the ClO_2_ production kinetics is also desirable.

## 5. Conclusions

Chlorine-dioxide-emitting, NaClO_2_-loaded PEO nanofibers were prepared via electrospinning. Chlorine dioxide generated from NaClO_2_-loaded PEO samples eliminated bacteria from the gas phase successfully in 5% (*v/v*) CO_2_ and RH > 95% in 24 h. The presence of the active ingredient did not result in major structural changes in the FTIR spectrum of the polymer. Chlorine dioxide production ability was measured from different weights of fibrous samples, and ClO_2_ yield was calculated. Exposing smaller fibrous samples to CO_2_ and humidity resulted in an increased ClO_2_ production ability and higher yield, while a higher mass of fibrous layer of nearly equal surface area could not produce proportionally higher amounts of chlorine dioxide. The concentration of CO_2_ did not alter the ClO_2_ production of the samples, while time of exposure increased the concentration of the generated chlorine dioxide. Fibrous NaClO_2_-loaded PEO samples present a substantial antimicrobial effect in conditions similar to exhaled air, as chlorine dioxide is formed in situ via the chlorite ion–acid reaction and emitted to the gas phase. One of the possible applications of the formulation discussed in our work may be the filters used in face masks, as the exhaled air is disinfected by chlorine dioxide generated from the fibers. Preventing the possible inhalation of ClO_2_ and providing one-way flow may be achieved by using breathing valves; however, toxicological evaluation is required. Electrospinning represents an effective and continuous method in the preparation of nanofibers. Industrial implementation and upscaling of the technique are also widely studied [38]. Electrospun, nanofiber-based formulations equipped with effective antimicrobial agents can serve as suitable tools in the protection against the wide spread of pathogens.

## Figures and Tables

**Table 1:** Yield of generated ClO_2_ by samples of different weights calculated from the theoretical ClO_2_ production of sodium chlorite in acidic environment.

m_sample_ (mg)	n_NaClO_2__ (mmol)	c(ClO_2_)_g_ (ppm)	c(ClO_2_)_g_ Theoretical (ppm) ^1^	Yield (%)
1	0.217	65.79	73.57	89.43
5	1.084	115.54	367.85	31.41
10	2.168	134.93	735.69	18.34
15	3.252	135.91	1103.54	12.32
20	4.336	141.16	1471.38	9.59
30	6.504	145.23	2207.07	6.58

^1^ Equations used to calculate the theoretical ClO_2_ concentration are shown in the Supplementary Materials.

## Data Availability

Not applicable.

## Supplementary Materials

The following supporting information can be downloaded at: https://www.mdpi.com/article/10.3390/nano12091481/s1, Equations (S1)–(S3): Chlorine dioxide production via the chlorite ion—acid system, Table S1: [ClO_2_]_g_ (M)/[ClO_2_]_aq_ (M) distribution constant (*K_θ_*) at different temperatures, Figure S1: Distribution constant at different temperatures along with the fitted polynomial equation [27,31].
