# Supplementary material for: Formulation of Chlorine-Dioxide-Releasing Nanofibers for Disinfection in Humid and CO2-Rich Environment
Source: Nanomaterials (Basel). 2022 Apr 27;12(9):1481. doi: 10.3390/nano12091481 (PMC9104377; doi:10.3390/nano12091481)
Supplement: Supplementary file 1 [file nanomaterials-12-01481-s001.zip › nanomaterials-1684002-supplementary.pdf]

Supplementary Materials

Formulation of Chlorine-Dioxide-Releasing Nanofibers for Disinfection in Humid and CO_2-Rich Environment

Barnabás Palcsó [1], Adrienn Kazsoki [1], Anna Herczegh [2], Ágoston Ghidán [3], Balázs Pinke [4], László Mészáros [4,5] and Romána Zelkó [1,*]

[1] University Pharmacy Department of Pharmacy Administration, Semmelweis University, Hőgyes Endre utca 7-9, H-1092 Budapest, Hungary; palcso.barnabas@pharma.semmelweis-univ.hu (B.P.); kazsoki.adrienn@pharma.semmelweis-univ.hu (A.K.)
[2] Department of Conservative Dentistry, Semmelweis University, Szentkirályi utca 47, H-1088 Budapest, Hungary; herczegh.anna@dent.semmelweis-univ.hu
[3] Institute of Medical Microbiology, Faculty of Medicine, Semmelweis University, Nagyvárad tér 4, H-1089 Budapest, Hungary; ghidan.agoston@med.semmelweis-univ.hu
[4] Department of Polymer Engineering, Faculty of Mechanical Engineering, Budapest University of Technology and Economics, Műegyetem rkp. 3, H-1111 Budapest, Hungary; pinke@pt.bme.hu (B.P.); meszaros@pt.bme.hu (L.M.)
[5] MTA–BME Research Group for Composite Science and Technology, Műegyetem rkp. 3, H-1111 Budapest, Hungary
* Correspondence: zelko.romana@pharma.semmelweis-univ.hu; Tel.: +36-1-2170927

The production of chlorine dioxide from sodium chlorite under acidic conditions can be described with the following equations [31]:

$$H^+ + ClO_2^- \rightleftharpoons HClO_2 \quad (1)$$

$$4\ HClO_2 \rightarrow 2\ ClO_2 + ClO_3^- + Cl^- + 2\ H^+ + H_2O \quad (2)$$

$$5\ HClO_2 \rightarrow 4\ ClO_2 + Cl^- + H^+ + H_2O \quad (3)$$

The first step is the protonation of chlorous acid with a pK_a of 1.72 (Equation 1). Chlorous acid then undergoes a self-decomposition reaction and chlorine dioxide is formed. There are two limiting cases of this process, and the stoichiometry can be calculated from the linear combination of Equation 2 and 3. First, an uncatalyzed reaction occurs, where the generated ClO_2:$HClO_2$ ratio is 1:2. In this reaction, chloride ion is produced, which acts a catalyzer and alters the process into Equation 3. Under ideal conditions, the ClO_2:$HClO_2$ ratio increases to 4:5. In our work we calculated the ClO_2 yield by Equation 3, assuming the ClO_2:$HClO_2$ ratio was 4:5.

The concentration of the produced chlorine dioxide in the gas phase was calculated from the absorbance measured in the water container placed into the glass bottles. Chlorine dioxide emitted from the fiber samples dissolves in the excess of water and a vapor-liquid equilibrium is reached. The distribution constant of ClO_2 between the two phases can be calculated from the concentrations measured in the gas and the aqueous phase (determined by Ishi) [27]. Using the data from Ishi's work we calculated the distribution constant regarding the parameters of our experiment.

Table S1. $[ClO_2]_g$ (M)/$[ClO_2]_{aq}$ (M) distribution constant (K_θ) at different temperatures.

Temperature (°C)	Distribution constant
0	0.0155
5	0.0189

10	0.0222
15	0.0267
20	0.0316
30	0.0428
40	0.0578

Figure S1 shows the K_{θ}–temperature curve along with the fitted polynomial equation. Using the equation, the distribution constant at 37°C can be calculated. At 37°C the $[ClO_2]_g$ (M)/$[ClO_2]_{aq}$ (M) ratio was 0.053. Further calculation to determine $[ClO_2]_g$ form $[ClO_2]_{aq}$ values is described in the manuscript.

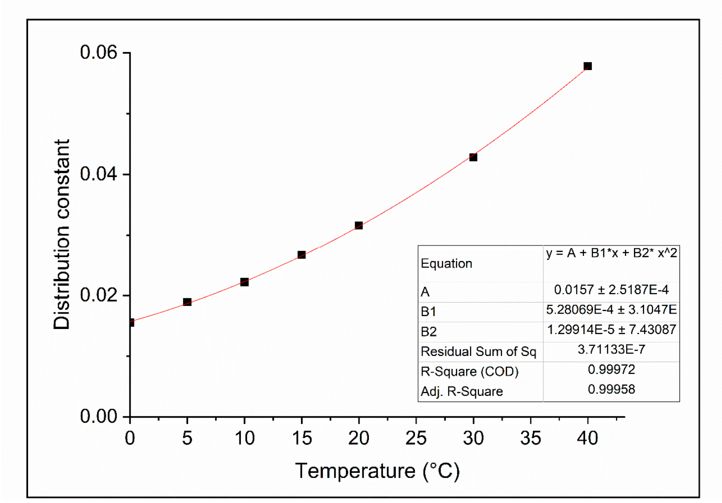

Figure S1. Distribution constant at different temperatures along with the fitted polynomial equation.
